# Sex differences in the metabolic effects of the renin-angiotensin system

**DOI:** 10.1186/s13293-019-0247-5

**Published:** 2019-07-01

**Authors:** Melissa C. White, Rebecca Fleeman, Amy C. Arnold

**Affiliations:** 1 Department of Comparative Medicine, Pennsylvania State University College of Medicine, 500 University Drive, Hershey, PA USA; 2 Department of Neural and Behavioral Sciences, Pennsylvania State University College of Medicine, 500 University Drive, Mail Code H109, Hershey, PA 17033 USA

**Keywords:** Gender, Insulin, Glucose, Energy balance, Obesity, Diabetes, Angiotensin

## Abstract

Obesity is a global epidemic that greatly increases risk for developing cardiovascular disease and type II diabetes. Sex differences in the obese phenotype are well established in experimental animal models and clinical populations. While having higher adiposity and obesity prevalence, females are generally protected from obesity-related metabolic and cardiovascular complications. This protection is, at least in part, attributed to sex differences in metabolic effects of hormonal mediators such as the renin-angiotensin system (RAS). Previous literature has predominantly focused on the vasoconstrictor arm of the RAS and shown that, in contrast to male rodent models of obesity and diabetes, females are protected from metabolic and cardiovascular derangements produced by angiotensinogen, renin, and angiotensin II. A vasodilator arm of the RAS has more recently emerged which includes angiotensin-(1-7), angiotensin-converting enzyme 2 (ACE2), *mas* receptors, and alamandine. While accumulating evidence suggests that activation of components of this counter-regulatory axis produces positive effects on glucose homeostasis, lipid metabolism, and energy balance in male animal models, female comparison studies and clinical data related to metabolic outcomes are lacking. This review will summarize current knowledge of sex differences in metabolic effects of the RAS, focusing on interactions with gonadal hormones and potential clinical implications.

## Introduction

The control of energy balance involves complex interactions between behavioral and physiological factors influencing energy intake, expenditure, and storage to maintain body weight and body composition within a homeostatic range [1]. As previously reviewed, several studies have shown sex differences in body composition, body fat distribution, and feeding behavior even in lean humans and rodent models [2]. Females have been shown to have higher adiposity and lower fat-free mass at any given body mass index (BMI) compared with males [3, 4]. Furthermore, females have more subcutaneous adipose tissue, particularly in abdominal and gluteofemoral regions, compared with a greater visceral adipose distribution in males [2]. These sex differences in adipose distribution have been linked with metabolic health, with females having a more favorable lipid and glucose metabolism profile compared with males [2].

Obesity results when food intake exceeds energy expenditure to promote excess energy storage in white adipose tissue [1]. Obesity is a global epidemic, affecting over 600 million individuals worldwide, which greatly increases risk for developing cardiovascular disease and type II diabetes [5, 6]. The energy imbalance seen in obesity is often accompanied by hypertension, chronic low grade systemic and adipose inflammation, macrophage infiltration in adipose tissue, and metabolic derangements such as hyperinsulinemia, hyperglycemia, hyperleptinemia, hyperlipidemia, insulin resistance, and glucose intolerance [7]. Sex differences in the obese phenotype are well recognized in experimental animal models and clinical populations [3, 4, 8]. Indeed, the prevalence of obesity has increased to a greater extent in women over the past decade [3, 4]. Despite this, premenopausal women are protected from the development of obesity-related metabolic and cardiovascular complications. Obese females, for example, generally have lower blood pressure, more tissue distributed to subcutaneous than pro-inflammatory visceral adipose tissue, smaller and more lipogenic and insulin-sensitive adipocytes, increased mass and metabolic activity of brown adipose tissue, higher levels of insulin-sensitizing hormones such as leptin and adiponectin, and greater peripheral insulin sensitivity when compared with obese males [3, 4, 8]. In addition, high-fat diet (HFD) feeding in male rodents increases pro-inflammatory M1 type macrophages in adipose tissue, increases percentage of pro-inflammatory T cells in the aorta and kidney, and reduces anti-inflammatory regulatory T cells (Tregs). In contrast, female HFD-fed rodents exhibit increases in anti-inflammatory M2 macrophages in adipose and maintain more Tregs in the aorta and kidney [8, 9]. These sex differences in macrophage polarization and T cell profile in response to HFD may contribute to metabolic and cardiovascular protection in females.

While still an active area of investigation, emerging evidence suggests that sex differences in obesity are, at least in part, attributed to hormonal mediators such as the renin-angiotensin system (RAS). This review will highlight recent developments in our understanding of sex differences in the metabolic effects of the RAS, including interactions with gonadal hormones and potential therapeutic implications for clinical populations. While not a focus of this review, sex differences in RAS components and actions have also been implicated in conditions closely related to metabolic function including aging [10], cardiovascular and renal diseases [11, 12], developmental programming [13], and hypertension [14–16].

### RAS pathways for metabolic regulation

#### Canonical RAS pathways

**Fig. 1:**
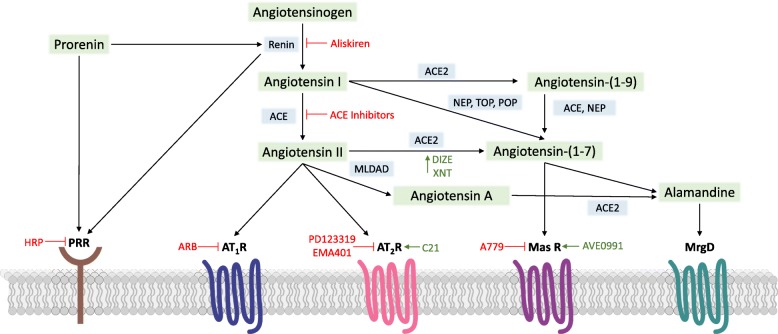
Simplified diagram of the renin-angiotensin system including sites of action for pharmacological agents targeting this hormonal system. A779, *mas* receptor antagonist [D-Ala^7^]-angiotensin-(1-7); ACE, angiotensin-converting enzyme; ARB, angiotensin receptor blocker; AT_1_R, angiotensin II type 1 receptor; AT_2_R, angiotensin II type 2 receptor; AVE0991, orally active *mas* receptor agonist; C21, compound 21 (AT_2_R agonist); DIZE, ACE2 activator diminazene aceturate; EMA401, AT_2_R agonist; HRP, decoy peptide for handle region of the prorenin prosegment; MasR, angiotensin-(1-7) *mas* receptor; MLDAD, mononuclear leukocyte-derived aspartate decarboxylase; MrgD, mas-related G protein-coupled receptor; NEP, neprilysin; POP, prolyl oligopeptidase; PRR, prorenin receptor; TOP, thimet oligopeptidase; XNT, ACE2 activator xanthenone

For over a century, the RAS has been recognized for its critical role in blood pressure regulation and the pathogenesis of cardiovascular diseases. Accumulating evidence suggests the RAS is also important in glucose homeostasis and energy balance, and that perturbations in this hormonal system are involved in development of metabolic diseases such as obesity and type II diabetes [17]. In the canonical RAS (Fig. 1), the enzyme renin is secreted into the circulation from renal juxtaglomerular cells in response to stimuli including increased sympathetic activity, local actions of nitric oxide and prostanoids, decreased renal afferent arteriole perfusion pressure, and decreased sodium chloride content in the macula densa of the renal distal tubules [18]. Renin acts upon angiotensinogen to form angiotensin (Ang) I, which is subsequently cleaved by Ang-converting enzyme (ACE) to form Ang II [19]. Ang II acts at cell surface type I G protein-coupled receptors (AT_1_R) to induce deleterious cardiovascular and metabolic effects including vasoconstriction, sympathetic activation, inflammation, oxidative stress, and insulin resistance [17, 20]. Ang II also binds cell surface type II receptors (AT_2_R) to counteract AT_1_R-mediated actions; although these receptors are more limited in tissue expression and affinity [21].

The Ang II-ACE-AT_1_R arm of the RAS has increased in complexity with recent findings including (1) Ang-(1-12), a C-terminally extended form of Ang I found in plasma and peripheral tissues, which is formed independent of renin and processed to Ang II [22]; (2) prorenin, which in addition to renin can bind the prorenin receptor (PRR) to induce non-proteolytic activation, generating Ang II in tissues and initiating Ang II-independent intracellular signaling [23]; (3) localization of RAS components in tissues (e.g., adipose, brain, kidney, skeletal muscle) [19], although the existence and independence of these local RAS systems from the circulation has been challenged [24]; (4) intracellular RAS capable of generating Ang II within cells (e.g., renal proximal tubule cells, neurons) or internalizing Ang II following cell surface receptor activation to elicit intracrine effects via AT_1_R-like nuclear receptors [25–27]; and (5) ACE-independent pathways for Ang II formation, particularly within tissues, involving actions of proteinases such as chymase, kallikrein, and cathepsin G [22].

#### Noncanonical RAS pathways

A counter-regulatory arm of the RAS has more recently emerged, which generally opposes actions of the Ang II-ACE-AT_1_R axis. As shown in Fig. 1, this noncanonical RAS is characterized by Ang-(1-7), which is formed from cleavage of Ang II by ACE2 or cleavage of Ang I by endopeptidases including neprilysin (NEP), prolyl oligopeptidase (POP), and thimet oligopeptidase (TOP) [28, 29]. Ang I can also be converted by ACE2 to Ang-(1-9) and subsequently cleaved by NEP or ACE to form Ang-(1-7). The actions of Ang-(1-7) at cell surface *mas* G protein-coupled receptors promote positive effects on blood pressure, glucose homeostasis, lipid metabolism, and energy balance [28]. While most physiological actions of Ang-(1-7) have been shown to require *mas* receptors, a few studies suggest heterodimerization and functional interplay between *mas* and AT_2_R [30]. Ang-(1-7) *mas* receptors may also heterodimerize with AT_1_R to competitively antagonize Ang II signaling [31]. Additionally, the endogenous heptapeptide alamandine was identified in 2013 in human blood and shown to differ from Ang-(1-7) only in its N-terminal amino acid [Ala^1^ versus Asp^1^ for Ang-(1-7)] [32]. As shown in Fig. 1, alamandine is formed through cleavage of Ang II to Ang A via mononuclear leukocyte-derived aspartate decarboxylase (MLDAD) with subsequent cleavage of Ang A via ACE2. Alamandine can also be formed via decarboxylation of Ang-(1-7) and binds mas-related G protein-coupled receptor D (MrgD) to elicit similar cardiovascular actions as Ang-(1-7) [33].

### Sex differences in metabolic effects of Ang II pathways

#### Angiotensinogen

Angiotensinogen, a glycoprotein serving as the main precursor of the RAS, is primarily liver-derived but is also expressed in numerous tissues including adipose [34]. In mice, adipose-derived angiotensinogen has been shown to contribute up to 30% of total circulating levels [35, 36]. Angiotensinogen gene expression in white adipose decreases with fasting and increases with increased nutrient availability or following exposure to long-chain fatty acids, glucocorticoids, cytokines, androgens, and hyperglycemia [34]. In obese animal models, adipose angiotensinogen is increased and correlates with systemic RAS activity and body mass [37]. In male mice, overexpression of angiotensinogen in adipose tissue results in hypertension, increased adiposity, insulin resistance, glucose intolerance, and reduced insulin-stimulated skeletal muscle glucose uptake [36, 38]. This increased adiposity and glucose intolerance is abrogated via ACE inhibition, suggesting Ang II-mediated effects [38]. In contrast, female mice with overexpression of adipose angiotensinogen exhibit normal insulin sensitivity and glucose tolerance [38].

Global deletion of angiotensinogen reduces body mass, adiposity, and circulating insulin and leptin levels in male mice [39]. Adipose-specific angiotensinogen deletion lowers resting blood pressure in male and female mice, with no effect on body weight, fat mass, or adipocyte size [35]. Despite lack of effect on body composition under resting conditions, adipose deletion of angiotensinogen attenuates HFD-induced metabolic dysfunction (e.g., weight gain, glucose intolerance, dyslipidemia) in male mice [40], as well as hypertension in male and female mice [41]. Finally, male transgenic rats with low brain angiotensinogen have reduced body mass and adiposity with improved glucose tolerance [42]. As summarized in Table 1, these overall findings suggest adipose-derived angiotensinogen contributes to hypertension and metabolic derangements and provide evidence for sex differences in the role of this RAS precursor in systemic glucose homeostasis.

**Table 1:** Sex differences in metabolic effects of the RAS in preclinical models

RAS component	Obesity	Dyslipidemia	Insulin sensitivity	Glucose tolerance	References
Angiotensinogen					
Males	↑, -	↑	↓	↓	[35, 36, 38–40, 42]
Females	-	UNK	-	-	[35, 38]
Renin					
Males	↑	↑	↓	↓	[43–50]
Females	-, ↑	-, ↑	-, ↓	-, ↓	[50, 51]
Prorenin					
Males	↑	↑	↓	↓	[52–55]
Females	↑	UNK	UNK	UNK	[56]
Angiotensin II					
Males	↑, ↓	↑	↓	↓	[17, 57–63]
Females	UNK	-	↓	↓	[64, 65]
ACE					
Males	↑	↑	↓	↓	[62, 66, 67]
Females	UNK	↑	↓	↓	[68, 69]
AT_1_ receptors					
Males	↑	-, ↑	↓	↓	[57, 58, 65, 70, 71]
Females	↑	UNK	↓	↓	[65, 72]
AT_2_ receptors					
Males	↓	↑, ↓	↓, ↑	↓, ↑	[61, 73–79]
Females	-	↓	↑	↑	[74, 80]
Angiotensin-(1-7)					
Males	↓	↓	↑	↑	[59, 81–90]
Females	UNK	UNK	UNK	UNK	
ACE2					
Males	↓	↓	-, ↑	↑	[91–97]
Females	UNK	UNK	UNK	UNK	
Mas receptors					
Males	-, ↓	↓	↑	↑	[97–99]
Females	-	UNK	UNK	UNK	[99]
Alamandine					
Males	UNK	UNK	UNK	UNK	
Females	UNK	UNK	UNK	UNK	

*ACE* angiotensin-converting enzyme, *ACE2* angiotensin-converting enzyme 2, *AT*_*1*_ angiotensin II type 1, *AT*_*2*_ angiotensin II type 2, *RAS* renin-angiotensin system, ↑ increases, ↓ decreases, - neutral effects, *UNK* information currently unknown

Despite strong evidence for upregulation in animal models, inconsistent findings have been reported for adipose angiotensinogen levels in human obesity [34]. Furthermore, clinical studies examining the impact of angiotensinogen on metabolic outcomes are lacking (Table 2). Polymorphisms of the angiotensinogen gene have been associated with insulin resistance in both sexes [100], increased risk for central obesity and dyslipidemia in hypertensive women with metabolic syndrome [101], and with visceral obesity and insulin resistance in obese Japanese women [102]. In addition, plasma angiotensinogen levels are correlated with waist circumference decline during weight loss in obese women [117].

**Table 2:** Sex differences in metabolic effects of the RAS in clinical opulations

RAS component	Obesity	Dyslipidemia	Insulin sensitivity	Glucose tolerance	References
Angiotensinogen					
Males	UNK	UNK	↓	UNK	[100]
Females	↑	↑	↓	UNK	[100–102]
Renin					
Males	↑	UNK	↓	↓	[103]
Females	↓, ↑	UNK	↓	↓	[103]
Prorenin					
Males	UNK	UNK	UNK	UNK	
Females	UNK	UNK	UNK	UNK	
Angiotensin II					
Males	↑	↑	↓	↓	[104–106]
Females	↑	↑	↓	↓	[104, 107]
ACE					
Males	-	↑	↓	↓	[108–110]
Females	-	↑	↓	↓	[101, 108, 110]
AT_1_ receptors					
Males	-	↑	↓	↓	[103, 105, 110–113]
Females	-	↑	↓	↓	[103, 105, 110, 112, 113]
AT_2_ receptors					
Males	UNK	UNK	UNK	UNK	
Females	↓	UNK	↑	↑	[114, 115]
Angiotensin-(1-7)					
Males	UNK	UNK	UNK	UNK	
Females	UNK	UNK	UNK	UNK	
ACE2					
Males	UNK	UNK	UNK	UNK	
Females	UNK	UNK	UNK	UNK	
Mas receptors					
Males	↑	UNK	UNK	UNK	[116]
Females	↑	UNK	UNK	UNK	[116]
Alamandine					
Males	UNK	UNK	UNK	UNK	
Females	UNK	UNK	UNK	UNK	

*ACE* angiotensin-converting enzyme, *ACE2* angiotensin-converting enzyme 2, *AT*_*1*_ angiotensin II type 1, *AT*_*2*_ angiotensin II type 2, *RAS* renin-angiotensin system, ↑ increases, ↓ decreases, - neutral effects, *UNK* information currently unknown

#### Renin and Prorenin

While renin, prorenin, and the PRR are established to play a role in cardiovascular regulation [118], their contribution to metabolic function is less understood. Renin is an aspartyl protease enzyme that is secreted from renal juxtaglomerular cells to initiate the RAS cascade to ultimately generate Ang II [18]. Additionally, renin is expressed in white adipose tissue, with higher levels in visceral than subcutaneous depots [34]. Sex differences in stimuli influencing renin release have been described with females generally having reduced sympathetic activation (particularly in obesity), increased renal nitric oxide synthesis, and a differential pattern of renal transporters influencing pressure natriuresis and electrolyte homeostasis [15, 119, 120]. Male mice with global deletion of the renin gene are lean due to enhanced energy expenditure, have improved insulin sensitivity, and are resistant to development of HFD-induced obesity [43]. These metabolic effects are reversed by systemic Ang II administration. Similarly, renin inhibition with aliskiren improves insulin sensitivity, skeletal muscle glucose uptake, glucose tolerance, and insulin secretion in male rodent models of hypertension, diabetes, obesity, and metabolic syndrome [44–48]. Conversely, male transgenic rodents overexpressing human renin are obese due to increased food intake and exhibit hyperglycemia, hyperinsulinemia, hyperlipidemia, and insulin resistance [49, 50]. This phenotype is not abrogated by ACE, renin, or prorenin inhibitors, suggesting Ang II-independent mechanisms. In contrast to male mice, female mice overexpressing human renin are protected from HFD-induced obesity [50]. Similar to males, however, renin inhibition with aliskiren improves glucose tolerance and insulin sensitivity in obese female Zucker rats [51]. Clinically, aliskiren is reported to lower blood pressure and improve whole-body insulin sensitivity in male and female hypertensive patients with metabolic syndrome [103].

Prorenin is an inactive precursor of renin, which contains a 43-amino acid prosegment covering the active cleft and is found in the circulation at concentrations at least tenfold higher than renin [23]. Renin and prorenin both bind the PRR to induce non-proteolytic activation, which generates Ang II in tissues and initiates Ang II-independent intracellular signaling [23]. In addition, PRR can be cleaved intracellularly by furin to secrete a soluble form of PRR in plasma and urine [121]. Increased non-proteolytic activation of prorenin has been observed in skeletal muscle and adipose tissue of male rat models of type II diabetes (fructose-fed and Otsuka Long-Evans Tokushima Fatty rats), in the absence of changes in PRR mRNA levels [52, 122]. In contrast, increased PRR mRNA has been shown in adipose tissue of high-fat/high-carbohydrate diet-induced obese male and female mice [53]. These disparate findings for PRR gene expression in metabolically-sensitive tissues may reflect the differences in sex, species, and use of obese versus diabetic models among these studies. Chronic treatment with HRP (a decoy peptide for the handle region of the prorenin prosegment that inhibits non-proteolytic PRR activation) attenuates weight gain, improves glucose tolerance, and reduces fasting insulin, leptin, triglyceride, and cholesterol levels in obese male rodents [52, 53]. Similarly, male mice with homozygous deletion of the PRR gene in adipocytes exhibit lower body mass and adiposity, higher lean mass, smaller visceral adipocytes, increased metabolic rate, and improved insulin sensitivity under normal diet conditions [54]. Male homozygous adipocyte PRR knockout mice are also resistant to HFD-induced obesity and glucose intolerance, despite elevated blood pressure and lipodystrophy [55]. Female heterozygous adipocyte PRR knockout mice do not exhibit altered body mass or adiposity under normal or HFD conditions, suggesting complete knockout of this gene is needed to influence adipose development [54]. Indeed, similar to males, female mice with homozygous adipose PRR deletion have reduced adiposity and are protected from HFD-induced obesity, despite increased blood pressure and renal cortical Ang II [56].

Clinically, a decrease in active renin and concomitant increase in prorenin is correlated with risk for diabetic nephropathy, retinopathy, and microvascular disease [123, 124]. While these studies included males and females, sex differences have not been explored. A few studies have examined adipose expression in clinical populations, with one study showing no difference in PRR gene expression or adipose depot distribution between lean and obese women [125]. Another study showed increased PRR and AT_1_R protein expression in subcutaneous adipose tissue of insulin-resistant postmenopausal non-diabetic obese women when compared with matched insulin-sensitive women [53]. Unfortunately, these studies did not include male subjects to determine sex-specific patterns of adipose PRR expression.

#### Angiotensin II, ACE, and AT_1_ receptors

The circulating and adipose Ang II-ACE-AT_1_R axis is activated in obesity and closely correlates with BMI, adiposity, and insulin resistance [17, 104]. Obesity-related hyperinsulinemia can stimulate endogenous Ang II production and subsequent AT_1_R stimulation [126]. Weight loss conversely decreases adipose angiotensinogen as well as circulating renin activity, Ang II, and aldosterone levels [117]. The overactivation of Ang II in obesity stimulates AT_1_R to promote hypertension, insulin resistance, and energy imbalance [17, 20]. While acute administration can improve insulin sensitivity in humans, chronic unregulated activation of Ang II pathways produces insulin resistance, glucose intolerance, and oxidative stress [17, 105].

Ang II promotes AT_1_R-mediated insulin resistance via multiple mechanisms including (1) aldosterone release, (2) direct uncoupling of intracellular insulin signaling pathways, (3) decreasing insulin-stimulated translocation of glucose transporter 4 (Glut4) to the cell membrane to subsequently reduce glucose uptake in peripheral tissues, (4) decreasing microvascular blood volume and flow to reduce glucose delivery, and (5) inhibiting insulin-mediated suppression of endogenous glucose production [17, 127]. In addition, Ang II increases inflammatory cytokine release, lipid transport, and triglyceride accumulation to promote lipotoxicity and impair insulin action in peripheral tissues such as pancreas, liver, and skeletal muscle [17]. RAS components including renin, ACE, and AT_1_R are also expressed in the pancreas and Ang II reduces pancreatic islet blood flow and induces oxidative stress and inflammation to impair pancreatic β-cell function and decrease glucose-stimulated insulin secretion [17]. For example, acute Ang II infusion decreases spontaneous and glucose-stimulated insulin secretion in healthy male subjects [106].

Ang II, ACE, and AT_1_R are expressed in white adipose tissue, with higher expression in visceral versus subcutaneous depots [34]. Ang II increases adipose inflammation and stimulates adipocyte differentiation and lipogenesis while inhibiting lipolysis [34]. In terms of energy balance, acute low-dose administration of Ang II reduces food intake and body weight in male rats [128]. More chronically, however, peripheral Ang II infusion promotes a positive energy balance in male rodents. In female atherosclerosis-prone mice, however, chronic Ang II infusion produces no effect on body mass or plasma cholesterol levels [64]. Systemic pharmacological blockade of Ang II activity with ACE inhibitors or angiotensin receptor blockers (ARBs), or global genetic deletion of AT_1_a receptors (AT_1a_R), protects male rodents against development of HFD-induced obesity and dyslipidemia by increasing energy expenditure and improving glucose tolerance and insulin sensitivity [57, 58, 129]. Similarly, ACE inhibitors and ARBs improve glucose homeostasis and reduce circulating fatty acid concentrations in obese female Zucker rats [65, 68]. This has also been shown in clinical studies, with the ARB irbesartan-reducing postprandial hypertriglyceridemia in male and female diabetic patients [130]. Enhancement of AT_1_R-associated protein (ATRAP; *Agtrap* gene), a local inhibitory protein promoting internalization of AT_1_R, in adipose tissue also attenuates HFD-induced obesity and insulin resistance in male mice [70].

Global deletion of the ACE gene protects male mice against obesity-related metabolic complications [66]. ACE gene polymorphisms have also been linked with increases in BMI and incidence of obesity in clinical populations [108, 109]. In contrast, male mice harboring an extra copy of the ACE gene have lower adiposity and body mass on HFD, and lower adiposity and increased energy expenditure after prolonged fasting. In contrast to peripheral effects, central Ang II infusion attenuates weight gain in lean and high-calorie cafeteria diet-fed male rats by decreasing food intake, increasing energy expenditure, and improving glycemic control [59, 60]. Furthermore, genetic deletion of AT_1a_R in either leptin receptor or agouti-related peptide-expressing cells within the hypothalamic arcuate nucleus in male and female mice results in failure to increase resting metabolic rate in response to HFD independent of blood pressure effects, with no gender differences reported [131]. This suggests opposing peripheral versus central Ang II actions on energy balance, as well as anatomical dissociation of cardiovascular versus metabolic control mechanisms. Importantly, most of these studies were performed in male rodents, with female comparisons lacking (Tables 1 and 2).

In male hypertensive rodent models, elevations in ACE activity are observed in the circulation, kidney, and heart [11]. In humans, serum ACE activity is also generally higher in adolescent and adult males versus females [132, 133]. One study also showed sex differences in RAS serum enzyme activity during healthy aging, with reduced ACE and aminopeptidase activity in older men compared with women [10]. Consistent differences in circulating Ang II levels have not been observed, with similar levels between males and females and between untreated and estrogen-treated females [11]. Discrepancies in sex differences in RAS components between humans and animal models may reflect that most clinical studies focus on systemic levels of RAS peptides, whereas animal studies concentrate on tissue levels of these peptides. Further investigation is needed to determine if there are sex differences in systemic and local Ang II concentrations and its effects on metabolic function.

Despite similar basal circulating levels, males appear to exhibit greater sensitivity to Ang II cardiovascular effects, with healthy men having greater pressor and renal vasoconstrictor responses to acute Ang II infusion compared with women [134]. Similarly, chronic Ang II infusion induces hypertension in male but not female rodents [135, 136], perhaps in part due to central estrogen protection shifting the balance from Ang II towards Ang-(1-7) pathways [137, 138]. Similar to findings for HFD exposure [9], recent studies have shown that immune cells may also underlie sex differences in Ang II-mediated hypertension [139]. For example, one study showed that chronic Ang II infusion in rats increases renal pro-inflammatory T cells in males while increasing anti-inflammatory Tregs in females [140]. Furthermore, while females gain more weight and adiposity on HFD, only males exhibit increased circulating Ang II levels and AT_1_R-mediated hypertension [141]. Male fructose-fed rats develop elevations in blood pressure associated with increased cardiac AT_1_R and ACE gene expression, with females protected from these derangements [142]. In contrast, despite having lower blood pressure and reduced renal ACE, female rats are not protected from the vascular and renal damage in early-onset diabetes [143]. A recent clinical study showed that in response to exogenous Ang II infusion, insulin resistance in women was associated with lack of response in heart rate variability and arterial stiffness, whereas men exhibited a protective increase in markers of cardiovagal function [107]. In summary, while females appear protected from hypertension resulting from activation of the Ang II-ACE-AT_1_R axis of the RAS, there is much less information on sex differences in metabolic outcomes (Tables 1 and 2).

#### AT_2_ receptors

While generally decreasing after birth, AT_2_R expression is increased in cardiovascular pathophysiological states as a potential compensatory mechanism to induce vasodilation to counteract AT_1_R-mediated actions [21]. Recent studies performed selectively in male rodents also implicate a role for AT_2_R in control of glucose homeostasis and energy balance (Table 1). The trophic actions of Ang II to promote adipocyte differentiation and lipogenesis in vitro are AT_2_R-mediated [144, 145]. Male mice with global AT_2_R gene deletion exhibit adipocyte hypotrophy and increased lipid oxidation, suggesting AT_2_R increases adipose cell mass and negatively regulates lipid utilization [73]. Similarly, global AT_2_R deletion in male mice protects against HFD-induced obesity, insulin resistance, glucose intolerance, and hypertension [73, 74]. In addition, AT_2_R deficiency in male mice protects against obesity induced by adipose angiotensinogen overexpression, as well as adipose tissue deletion during prolonged fasting [75, 146]. These anti-obesity effects are associated with reduced food intake and increases in energy expenditure, lipid oxidation, plasma thyroid levels, and urinary estrogen levels. This suggests that AT_2_R suppresses resting metabolic rate to contribute to obesity in male rodents. Consistent with this, one study showed that AT_2_R activation reduces differentiation and thermogenic capacity of subcutaneous white adipocytes to suppress resting metabolic rate in male transgenic mice with brain RAS activation [147]. Other studies, however, have shown AT_2_R activation reduces adiposity, improves glucose uptake and insulin sensitivity, and increases nitric oxide-mediated microvascular perfusion to enhance insulin delivery and action in skeletal muscle of male rodent models with diabetes and metabolic syndrome [61, 76–79]. AT_2_R activation has also been shown to improve pancreatic islet insulin biosynthesis and secretion in vitro and in vivo in diabetic male rats*,* in part by protecting β-cells from oxidative stress and apoptosis [148]. These findings show inconsistent effects of AT_2_R deletion versus activation on metabolic outcomes in male rodent models.

The AT_2_R gene is located on the X chromosome [11], with accumulating evidence supporting sex-specific metabolic actions of this receptor. In contrast to male mice, global AT_2_R deletion exacerbates HFD-induced weight gain, adiposity, hyperinsulinemia, glucose intolerance, and estrogen depletion in female mice [74]. The physiological mechanisms by which AT_2_R contributes to sex differences in obesity susceptibility in mice remain unclear but may involve differential effects on estrogen levels. Similar to genetic deletion in mice, a polymorphism in the AT_2_R gene (A/C^3123^) is associated with modest increases in BMI and hemoglobin A1C levels in healthy Japanese women [114, 115]. Conversely, AT_2_R activation attenuates HFD-induced weight gain, adiposity, and hyperinsulinemia in female mice independent of urinary estrogen levels [80]. These findings suggest that AT_2_R may be metabolically protective, particularly in females (Tables 1 and 2).

#### ACE inhibitors and angiotensin receptor blockers

Pharmacological blockade of Ang II formation and AT_1_R-mediated actions with ACE inhibitors and ARBs (Fig. 1), respectively, is commonly used for hypertension treatment in obese and type II diabetic patients due to their positive metabolic profile. In addition to cardioprotection, these therapies improve insulin sensitivity via several mechanisms including enhancement of bradykinin-nitric oxide pathways, upregulation of insulin signaling pathways, vasodilation to enhance glucose delivery, and improved Glut4 trafficking to increase whole-body glucose disposal [58, 127]. These therapies also have protective effects on pancreatic β-cells such as increasing islet blood flow and reducing oxidative stress to increase glucose-stimulated insulin release [58]. ACE inhibitors and ARBs reduce incidence of new-onset diabetes in large randomized trials in hypertension, chronic heart failure, and patients at high risk for cardiovascular events [110]. Furthermore, both ACE inhibitors and ARBs protect against HFD-induced weight gain, dyslipidemia, insulin resistance, and glucose intolerance in male rodents [62, 67, 149]. The anti-obesity effects of ARBs in mice may involve induction of thermogenic beige adipocytes to increase energy expenditure [150]. Clinical studies examining chronic effects of RAS blockade have shown improved dyslipidemia but inconsistent results for energy balance, with some studies showing weight loss and others showing no effect on body mass [151–153].

There is limited data on the influence of sex on RAS blockade efficacy. In terms of hypertension control, one meta-analysis revealed that sex-specific outcome data were only reported in 43% of clinical trials reviewed, with ACE inhibitors and ARBs showing a small increase in cardiovascular benefit in men versus women [154]. Reduced blood pressure lowering effects of ACE inhibition in females has also been supported in animal studies [155], with ARBs potentially providing more benefit in females. One study showed sex differences in pancreatic blood flow responses to Ang II blockade in diabetic rats, with ACE inhibitors increasing serum insulin only in male rats, and ARBs increasing pancreatic and islet blood flow only in female rats [156]. ARBs also appear metabolically protective in obese female rats to prevent obesity-related metabolic and ovulatory defects [72]. While clinical trials examining incidence of new-onset diabetes with ACE inhibitors and ARBs-enrolled males and females [110], subgroup analysis was only performed in a few of these studies and showed no impact of sex on cardiovascular and metabolic protection [157–159]. Similarly, a retrospective study showed no association of sex with the blood glucose-lowering effects of ARBs in hypertensive Japanese patients [160]. These findings highlight the need for further animal and clinical studies examining the impact of sex on effects of RAS blockade in terms of metabolic outcomes.

### Sex differences in metabolic effects of Ang-(1-7) pathways

#### Angiotensin-(1-7)

In contrast to Ang II, activation of Ang-(1-7) pathways promote positive metabolic effects in male rodent models of obesity, diabetes, and cardiometabolic syndrome. There is evidence from both in vitro and in vivo experiments to support a positive influence of Ang-(1-7) on intracellular insulin signaling pathways by increasing phosphorylation of insulin receptor substrate 1 and Akt in the heart, liver, skeletal muscle, and adipose tissues [81]. Ang-(1-7)-mediated Akt activation influences downstream modulators of glucose metabolism including endothelial nitric oxide synthase, AS160 (negative regulator of Glut4 translocation), and glycogen synthase kinase-3β (proline-directed serine-threonine kinase inactivating glycogen synthase). Ang-(1-7) also acts via *mas* receptors to increase basal and/or insulin-stimulated glucose uptake in cultured adipocytes, hepatocytes, and cardiomyoctes in male rodents [161–163]. Importantly, Ang-(1-7) reverses Ang II-mediated inhibition of insulin signaling and glucose transport activity in insulin-sensitive tissues of male rodents [81].

Chronic targeting of Ang-(1-7) improves glucose homeostasis and insulin action in male rodents. In male fructose-fed rats, chronic peripheral or central Ang-(1-7) infusion lowers blood pressure, improves insulin sensitivity and glucose tolerance, reduces insulin levels, and increases insulin signaling in the liver, skeletal muscle, and adipose tissues [82–84]. Similarly, male transgenic rats with elevated circulating Ang-(1-7) levels are lean and have improved insulin sensitivity and glucose tolerance in part due to enhanced adipocyte glucose uptake [164]. In HFD-induced obese male mice, plasma Ang-(1-7) is reduced and chronic peripheral restoration of this hormone reverses whole-body insulin resistance by enhancing insulin-stimulated skeletal muscle glucose uptake via enhanced Glut4 translocation independent of body composition or blood pressure [85]. Acute intravenous Ang-(1-7) also enhances insulin action and improves insulin sensitivity in lean male rats by enhancing skeletal muscle glucose uptake [86, 165]. Administration of orally active Ang-(1-7) improves hyperglycemia, hyperinsulinemia, and insulin resistance in male diabetic rats [163]. Ang-(1-7) also improves insulin secretion in vitro and in vivo, regulates development of pancreatic endocrine cells, and protects pancreatic β-cells by attenuating islet endothelial cell dysfunction, reducing β-cell dedifferentiation, and improving microcirculation [87, 91, 166, 167]. In summary, in male obese and diabetic rodents, Ang-(1-7) improves insulin signaling, insulin-stimulated glucose uptake via Glut4, and insulin secretion.

In addition, Ang-(1-7) improves energy balance and lipid metabolism in male rodents. Chronic peripheral Ang-(1-7) infusion induces brown adipocyte differentiation to increase thermogenesis and attenuate weight gain in HFD-induced obese male mice [88]. Chronic central Ang-(1-7) infusion also produces anti-obesity effects in male rats, although potency of these effects is lower than Ang II [59]. Mechanistically, anti-obesity effects of peripherally administered Ang-(1-7) have been linked with regulatory effects on lipid metabolism pathways, particularly in adipose tissue. Noncanonical RAS components such as Ang-(1-7), ACE2, and *mas* receptor are expressed in adipose tissue. Ang-(1-7) reduces plasma total cholesterol and triglyceride levels [82, 89, 90], decreases lipid accumulation in tissues, protects against adipose inflammation, and preserves insulin signaling in adipocytes in male rodents [88, 168]. The beneficial adipose effects of Ang-(1-7) may involve anti-inflammatory effects as well as modulation of sirtuins or other proteins involved in lipid metabolism (monoglyceride lipase), redox processes (carbonic anhydrases), or energy transduction (annexin A2) [168, 169].

While accumulating evidence shows Ang-(1-7) has beneficial metabolic effects in male rodents, female comparison studies are lacking (Table 1). In hypertensive rats, females have greater circulating and renal Ang-(1-7) levels compared with males, although these sex differences appear strain-specific [25]. One study also showed sex-dependent circulating Ang-(1-7) levels in HFD-induced obese mice [141]. Obese female mice had higher circulating Ang-(1-7) and adipose ACE2 levels and were protected from development of hypertension, despite having more body mass and adiposity compared with males. Ovariectomy or chronic administration of the *mas* receptor antagonist [D-Ala^7^]-Ang-(1-7) [A779] elevated nocturnal blood pressure in these obese female mice, with no information on metabolic outcomes, suggesting estrogen and Ang-(1-7) interactions are important in this cardiovascular protection [141].

It is unclear if sex differences similarly exist for Ang-(1-7) levels in clinical populations. While one study reported higher plasma Ang-(1-7) in healthy young adult males versus females [170], another study found that females have higher levels of this hormone in a healthy adult cohort [171]. This could reflect differences in use of protease inhibitors during blood sample collection as well as heterogeneity in terms of geographical location, age, and racial demographics. In the latter study, Ang-(1-7) positively correlated with diastolic blood pressure in females, and with endothelial function in both sexes [171]. An additional study found urinary Ang-(1-7) is higher in females in a normotensive Afro-Caribbean population and positively correlates with systolic blood pressure but not BMI or waist circumference [172]. There were no sex differences in plasma Ang-(1-7), plasma renin activity, or plasma or urinary Ang II levels in this study, consistent with differential processing mechanisms for individual RAS components. During healthy aging, there are no differences in Ang-(1-7)-forming enzyme activities (e.g., ACE2, neprilysin) between men and women [10]. In summary, there are few studies exploring sex differences in Ang-(1-7) in animal models and clinical populations, with most studies focused on differences in levels of this hormone related to cardiovascular regulation. Additional studies examining Ang-(1-7) levels in obesity and type II diabetes, and potential sex differences in terms of metabolic actions, are needed.

While Ang-(1-7) is an attractive therapeutic target for obesity and type II diabetes, this approach is currently limited by the short half-life of the hormone. Oral formulations and stable analogs of Ang-(1-7) are being tested in animal models [173–175]; however, presently, there are limited clinical studies. The published clinical studies to date have largely focused effects of intra-arterial or intravenous infusion on cardiovascular outcomes such as vasodilation and blood pressure in healthy subjects and patients with essential hypertension, heart failure, and obesity [29, 176, 177]. Of interest, therapies blocking Ang II activity such as ACE inhibitors and ARBs shift the balance of the RAS to increase Ang-(1-7) levels [29]. While generally attributed to reduced Ang II formation, studies in male rodents have shown that cardiovascular and metabolic effects produced by these therapies may result from this endogenous Ang-(1-7) production [178–180]. Furthermore, while ACE inhibitors and ARBs remain mainstays of cardiovascular therapy, a recent meta-analysis provided evidence for an approximate 11% pooled incidence of cough among randomized, controlled clinical trials with ACE inhibitors [181]. These drugs can also rarely cause angioedema related to production of kinins [182]. Direct targeting of Ang-(1-7) may therefore represent an advantageous approach to avoid these side effects.

#### ACE2

ACE2 is a monocarboxypeptidase that preferentially removes carboxy-terminal amino acids from substrates including Ang II, Ang I, and apelin [183]. While circulating levels are low to undetectable under normal conditions, ACE2 expression and activity is found in multiple tissues including heart, kidney, liver, skeletal muscle, adipose, and pancreas [183]. ACE2 expression is upregulated in the serum, kidney, pancreas, and liver of male and female diabetic rodents suggesting a compensatory protective mechanism [143, 184, 185]. Urinary levels are also elevated in diabetic male mice due to increased proteolytic cleavage of the extracellular catalytic domain of ACE2 [186]. This ACE2 shedding positively correlates with proteinuria, glucose and triglyceride levels. Serum ACE2 activity is also elevated in patients with type I diabetes mellitus and microalbuminuria, and urinary ACE2 is elevated in diabetic renal transplant patients [105].

In preclinical models, the role of ACE2 in glucose homeostasis and energy balance has been limited to studies in males (Table 1). Under normal diet conditions, one study showed lack of a significant metabolic phenotype in male mice with global deletion of the ACE2 gene [92], while another study showed decreased glucose-stimulated first-phase insulin secretion and progressively impaired glucose tolerance with ACE2 deletion [93]. When challenged pharmacologically with Ang II or physiologically with either HFD or high-fat high-sucrose diets, male ACE2 knockout mice exhibit greater impairments in insulin sensitivity, glucose tolerance, and glucose-stimulated insulin secretion when compared with male control mice [91, 92]. Obese male ACE2 knockout mice also have reduced mass and proliferation of β-cells [94], and higher percentage of dedifferentiated β-cells [91], suggesting ACE2 is protective to pancreatic function.

Pharmacological approaches to chronically increase ACE2 have been explored for obesity, diabetes, and hypertension in animal models. The orally active ACE2 activator diminazene aceturate (DIZE) reduces body mass and adiposity, improves plasma lipid profile, and decreases adipogenesis markers in lean and obese male rodents [95, 96]. Furthermore, human recombinant ACE2 administration and ACE2 activators (e.g., DIZE, xanthenone) protect against diabetes-induced complications including cardiac and renal dysfunction and retinopathy in male mice [187, 188]. Human and mouse recombinant ACE2 has also been shown to protect against Ang II-induced hypertension in male mice [189, 190]. Pancreatic-specific adenoviral ACE2 overexpression has no effect on insulin sensitivity but improves glycemia and glucose tolerance in diabetic male mice [97]. The mechanism underlying this improved glycemic control may involve increased β-cell proliferation and glucose-stimulated first-phase insulin secretion and decreased islet apoptosis. ACE2 overexpression also increases basal and insulin-stimulated glucose uptake in isolated adipocytes and hepatocytes from male rodents, in part, by improving insulin signaling and reducing oxidative stress [161, 162]. A limitation of many of these studies remains the unclear conclusions as to whether the beneficial metabolic effects of ACE2 activation in male mice reflects reduced Ang II levels versus increased Ang-(1-7) formation, or a combination of these mechanisms.

The ACE2 gene is located on the X chromosome, with females generally having higher ACE2 activity [11]. Only one study to date has reported sex-specific differences in ACE2 expression and activity in mice, with chronic HFD administration reducing renal ACE2 in males and increasing adipose ACE2 in females [141]. The increased adipose ACE2 in obese female mice was reversed by ovariectomy suggesting estrogen-mediated effects. In addition, global deletion of ACE2 augments HFD-induced obesity hypertension in male mice, and induces obesity hypertension in female mice, by increasing the circulating Ang II:Ang-(1-7) balance [141]. Interestingly, these effects appear independent of body weight, as ACE2 knockout mice had attenuated weight gain during HFD feeding compared with wild-type littermate controls. Similar to males [189, 190], recombinant ACE2 protects female mice against hypertension induced by Ang II infusion or transgenic overexpression of renin [191]. Given findings for sex-specific ACE2 expression in obese and diabetic rodent models, exploring sex differences in the metabolic and cardiovascular therapeutic potential for ACE2 remains a critical area for research.

#### Mas receptors

In addition to mediating effects of exogenous Ang-(1-7), accumulating evidence suggests endogenous Ang-(1-7) activates *mas* receptors to influence resting metabolic function. In support of this, pharmacological blockade of *mas* receptors with A779 reduces insulin sensitivity in diabetic male mice [97] and impairs adipocyte responsiveness to antilipolytic effects of insulin [192]. Additionally, gene silencing of *mas* receptors in human subcutaneous preadipocytes reduces adipogenic markers [192]. Global deletion of *mas* receptors in male FVB/N mice results in a metabolic syndrome phenotype characterized by increased adiposity, hyperglycemia, hyperinsulinemia, increased circulating and skeletal muscle triglycerides, insulin resistance, glucose intolerance, and reduced glucose uptake and Glut4 levels in adipose tissue [98]. In contrast, *mas* receptor deletion in male and female C57Bl/6J mice has no effect on body mass or composition under control diet or HFD conditions [99]. These disparate findings are likely due to differences in background strains but could reflect length of diet administration and age of mice at time of study. Interestingly, a nonpeptide orally active *mas* receptor agonist, AVE0991, has been developed with beneficial cardiovascular effects shown in hypertensive and diabetic male rodents [174, 193]. One study has shown glucose and lipid lowering effects of AVE0991 in diabetic male rats [175]; however, this compound has not yet been evaluated in humans.

A handful of studies have described sex differences related to *mas* receptors, primarily related to cardiovascular function. One study demonstrated that obese female mice have elevated plasma Ang-(1-7) levels and are protected from hypertension [141]. This cardiovascular protection was eliminated following chronic systemic blockade of *mas* receptors with A779 suggesting Ang-(1-7)-mediated effects [141]. Similarly, global *mas* receptor deletion promotes obesity hypertension in female but not male mice, with no effect on body composition in either sex [99]. In summary, while *mas* receptors appear important for cardioprotection in female rodents [99, 141], there are limited data on sex differences related to metabolic outcomes in preclinical models (Table 1). As summarized in Table 2, there is limited information in clinical populations, with one study showing that single nucleotide polymorphisms in the *mas* receptor gene may contribute to obesity risk in a Chinese population-based cohort [116].

#### Alamandine

Alamandine is a recently discovered component on the noncanonical arm of the RAS, which acts at MrgD receptors to produce vasodilatory and cardioprotective effects similar to Ang-(1-7) in animal models [33]. A recent study shows that mice with global deletion of MrgD receptors develop dilated cardiomyopathy at an early age [194]. This cardiomyopathy was seen to a similar extent in male and female mice, suggesting a sex-independent role for endogenous alamandine in cardiac function. In terms of metabolic function, one study showed alamandine decreases leptin secretion and expression from visceral white adipocytes in vitro and reduces circulating leptin levels in vivo, in male rats [195]. These effects were mediated via activation of mitogen-activated protein kinase pathways and were opposite to effects of Ang-(1-7) to increase leptin secretion and expression. There are currently no preclinical or clinical studies exploring the role of alamandine in glucose homeostasis, lipid metabolism, or energy balance (Tables 1 and 2). Given its recent discovery, an opportunity exists for research exploring effects of alamandine on metabolic function and related sex differences.

### Interactions of the RAS with sex hormones

As recently reviewed, premenopausal women have a more favorable lipid and glucose metabolism profile, more adipose tissue distributed to subcutaneous depots, and lower blood pressure compared with men, with protection largely attributed to the presence and positive metabolic and cardiovascular actions of estrogen [2, 196]. Weight gain and blood pressure are enhanced during aging and particularly following menopause, with the incidence of obesity reaching ~ 65% in women over the age of 40 in the USA [197]. Replacement of estrogen may be beneficial for metabolic outcomes as a meta-analysis of randomized controlled trials found that hormone replacement therapy improves fat-free mass and insulin sensitivity and decreases visceral fat and cholesterol levels independent of blood pressure effects, route of administration, or whether a progestin component was included [198].

Accumulating evidence suggests that several components of the RAS are regulated by sex hormones (Table 3), as well as influenced by hormone replacement therapies. The angiotensinogen gene has an estrogen-responsive element, with females generally having higher circulating levels compared with males [199]. In rats, estrogen increasing circulating levels and hepatic gene expression of angiotensinogen [200]. Conversely, in normal and hypertensive rats, angiotensinogen gene expression is decreased in liver and kidney following castration and increased with exogenous testosterone administration [201, 202]. Similar to preclinical models, oral estrogen replacement therapy exerts a positive regulatory influence on angiotensinogen secretion in postmenopausal women irrespective of hypertensive status, with no information on androgen effects on this precursor in clinical populations [203–205].

**Table 3:** Regulatory interactions between the RAS and sex hormones

RAS component	Estrogen	Androgen	References
Angiotensinogen			
Preclinical	↑	↑	[199–202]
Clinical	↑	UNK	[203–205]
Prorenin			
Preclinical	UNK	↑	[206, 207]
Clinical	↓	UNK	[133]
Renin			
Preclinical	-	↑	[201, 208, 209]
Clinical, PRC	↓	UNK	[133, 203, 205]
Clinical, PRA	↑	UNK	[204, 210, 211]
ACE			
Preclinical	↓	↑	[208, 212, 213]
Clinical	↓	↑	[205, 214, 215]
Angiotensin II			
Preclinical	↑, ↓	-	[208, 216, 217]
Clinical	↑	UNK	[204, 205, 211, 214]
AT_1_R			
Preclinical	↓	↑	[212, 216, 218–221]
Clinical	UNK	UNK	
AT_2_R			
Preclinical	↑	↓	[219, 222, 223]
Clinical	UNK	UNK	
Angiotensin-(1-7)			
Preclinical	↑	UNK	[141, 208]
Clinical	↑	UNK	[171]
ACE2			
Preclinical	↑	-	[141, 224, 225]
Clinical	UNK	UNK	
Mas receptor			
Preclinical	UNK	UNK	
Clinical	UNK	UNK	
Alamandine			
Preclinical	UNK	UNK	
Clinical	UNK	UNK	

*ACE* angiotensin-converting enzyme, *ACE2* angiotensin-converting enzyme 2, *AT*_*1*_*R* angiotensin II type 1 receptor, *AT*_*2*_*R* angiotensin II type 2 receptor, *PRA* plasma renin activity, *PRC* plasma renin concentration, *RAS* renin-angiotensin system, ↑ increases, ↓ decreases, - neutral effects. *UNK* information unknown

In terms of prorenin, testosterone increases plasma levels in mice and rats [206, 207], with no information on estrogen effects. In clinical populations, women taking estrogen replacement therapy have lower plasma prorenin levels compared with men and women not taking estrogen replacement therapy [133], with no information on androgen effects. In hypertensive rats, estrogen appears to have no influence on plasma renin concentration; however, anti-androgen therapy decreases renin suggesting a positive regulatory relationship with testosterone [201, 208, 209]. In clinical populations, plasma renin concentration is lower in postmenopausal women compared with men, with levels particularly lower in women taking estrogen replacement therapy [133, 203]. While most clinical studies support that estrogen decreases plasma renin concentration [133, 203, 205], estrogen replacement therapy often increases plasma renin activity in postmenopausal women perhaps reflecting higher levels of angiotensinogen substrate [204, 210, 211]. Additionally, there are conflicting views on the impact of oral versus transdermal estrogen replacement therapy on renin. While one study showed that both routes of administration decrease plasma renin activity [203], another showed that oral administration increases plasma renin activity with no effect of transdermal administration [214].

In preclinical models, estrogen has been shown to decrease serum and tissue ACE expression, tissue AT_1_R expression and density, and aldosterone production, while testosterone conversely increases ACE activity and tissue AT_1_R expression [208, 212, 213, 216, 218–221, 226]. The influence of estrogen on circulating Ang II levels is less clear, with one study showing a decrease following estrogen replacement therapy in hypertensive rats [208], while another study showed an increase following estrogen treatment in normal rats [217]. One study has also shown no effect of gestational exposure to testosterone on circulating Ang II levels in rats [216]. In clinical populations, ACE activity is similar among age-matched women and men, regardless of menopausal status [203]. Plasma Ang II levels are also similar in normotensive premenopausal women compared with men [227]. Oral estrogen replacement therapy decreases circulating ACE activity and increases Ang II levels in postmenopausal women, with no effect of transdermal administration on these RAS components [204, 205, 214, 215].

There is currently limited data on interactions of gonadal hormones with counter-regulatory RAS components, with research mostly limited to animal models. Estrogen upregulates AT_2_R binding and expression in adrenal and renal tissues in male and female rats, suggesting a positive regulatory interaction [219, 222]. Reciprocally, AT_2_R stimulation increases ovarian estrogen production and stimulates ovulation and oocyte maturation in rabbits [228]. Testosterone conversely downregulates AT_2_R gene and protein expression levels in rat aorta [223]. Circulating Ang-(1-7) levels appear higher in obese female mice and in healthy women when compared with males [141, 171]. Estradiol administration increases circulating Ang-(1-7) levels and renal ACE2 gene expression in hypertensive rats and amplifies the vasodilator properties of Ang-(1-7) in ovariectomized rats [208, 224, 229]. Additionally, plasma Ang-(1-7) levels and adipose ACE2 activity are reduced by ovariectomy in obese female mice [141]. Testosterone has no effect on ACE2 mRNA in cultured adipocytes, or on testicular ACE2 activity in lean rats [225]. Overall, these data suggest that in animal models, estrogen shifts the balance of the RAS from the deleterious Ang II-ACE-AT_1_R axis to the beneficial Ang-(1-7)-ACE2-*mas* receptor axis, an effect which could promote positive cardiometabolic effects.

## Conclusions

The identification of sex-specific mechanisms underlying metabolic effects of the RAS, as well as beneficial effects of therapies targeting the RAS, remains an active area of research. Sex differences in expression, activity, and tissue responsiveness of several RAS components are apparent, with estrogen downregulating Ang II and upregulating Ang-(1-7) pathways [11]. In animal models of obesity, females appear to maintain circulating Ang-(1-7) levels [141] and are protected from hypertension and metabolic complications induced by angiotensinogen, renin, angiotensin II, and AT_1_R activation [38, 50, 142]. While inconsistent effects are observed in males, stimulation of counter-regulatory AT_2_R appears metabolically protective in female rodents [80, 114, 115, 147]. Activation of Ang-(1-7) pathways is also an attractive target to improve glucose homeostasis, lipid metabolism, and energy balance in male rodent models of obesity [28]. While development of pharmacotherapies activating Ang-(1-7) pathways may broaden therapeutic potential for RAS targeting, few studies have been performed in female animal models, with no clinical data in either sex supporting efficacy on metabolic outcomes. This underlines the important issue of translating findings related to sex differences in RAS therapies from experimental animal models to clinical practice. Current therapeutic recommendations for metabolic and cardiovascular complications in obesity are not specific to men versus women, even though sex differences in RAS pathways are evident. Furthermore, while large controlled clinical trials for RAS inhibition enroll both men and women, few studies have assessed sex-specific effects on cardiovascular and metabolic outcomes. These collective findings illustrate the critical need for additional mechanistic and clinical research to determine the impact of sex on metabolic effects of the RAS.

## Data Availability

Not Applicable

## Abbreviations

A779: [D-Ala^7^]-angiotensin-(1-7)
ACE: Angiotensin-converting enzyme
ACE2: Angiotensin-converting enzyme 2
Ang: Angiotensin
ARB: Angiotensin receptor blocker
AT_1_R: Angiotensin II type 1 receptor
AT_2_R: Angiotensin II type 2 receptor
AVE0991: Orally active *mas* receptor agonist
BMI: Body mass index
DIZE: Diminazene aceturate
Glut4: Glucose transporter 4
HFD: High-fat diet
MLDAD: Mononuclear leukocyte-derived aspartate decarboxylase
MrgD: Mas-related G protein-coupled receptor
PRR: Prorenin receptor
RAS: Renin-angiotensin system
